# I Am Not Invisible: The Impact of Age Discrimination in the Workplace

**DOI:** 10.1093/geroni/igaa057.080

**Published:** 2020-12-16

**Authors:** Jasmin Tahmaseb McConatha, Frauke Schnell, Lauren Stricker, Jacqueline Magnarelli

**Affiliations:** West Chester University, West Chester, Pennsylvania, United States

## Abstract

Ageism and age stereotypes are widespread. They shape the lived experiences of older workers. This presentation focuses on the results of responses to an online survey exploring the impact of ageist treatment in the workplace. The results of online surveys from 113 teachers over the age of 50 indicated that ageist treatment is widespread. An analysis of open ended questions addressing the stressful impact of being victimized by ageism indicated that feeling invisible, isolated, and helpless are the three most common responses to ageist treatment in the workplace. Being victimized by ageism presents a threat to older workers sense of self and feelings of competence. The cultivation hypothesis suggests that in technologically advanced societies such as the United States, people often rely on the media as a primary source of cultural information. Media images tend to depict older adults in ways that maintain and create ageist stereotypes. Our research suggests that the framing of media content significantly influences the self-worth of older workers. In this presentation, we discuss examples of ageism in the workplace, the family, and the media, and discuss ways of combating biased and discriminatory treatment. Based on our ongoing research, we make suggestions for ways of responding to and coping with ageist treatment.

